# Parent and clinician perceptions and recommendations on a pediatric cancer pain management app: A qualitative co-design study

**DOI:** 10.1371/journal.pdig.0000169

**Published:** 2023-11-29

**Authors:** Lindsay A. Jibb, Surabhi Sivaratnam, Elham Hashemi, Charlene H. Chu, Paul C. Nathan, Julie Chartrand, Nicole M. Alberts, Tatenda Masama, Hannah G. Pease, Lessley B. Torres, Haydee G. Cortes, Mallory Zworth, Susan Kuczynski, Michelle A. Fortier

**Affiliations:** 1 Lawrence S. Bloomberg Faculty of Nursing, University of Toronto, Toronto, Canada; 2 Child Health Evaluative Sciences, Hospital for Sick Children, Toronto, Canada; 3 Temerty Faculty of Medicine, University of Toronto, Toronto, Canada; 4 KITE-Toronto Rehabilitation Institute, University Health Network, Toronto, Canada; 5 Institute of Health Policy Management and Evaluation, University of Toronto, Toronto, Canada; 6 Division of Hematology/Oncology, Hospital for Sick Children, Toronto, Canada; 7 Faculty of Health Sciences, University of Ottawa, Ottawa, Canada; 8 Research Institute, Children’s Hospital of Eastern Ontario, Ottawa, Canada; 9 Department of Psychology, Concordia University, Montreal, Canada; 10 Sue and Bill Gross School of Nursing, University of California Irvine, Irvine, California, United States of America; 11 Department of Pediatric Psychology, Children’s Hospital of Orange County, Orange, California, United States of America; 12 UCI Center on Stress and Health, University of California Irvine, Irvine, California, United States of America; 13 Ontario Parents Advocating for Children with Cancer, Toronto, Canada; Iran University of Medical Sciences, IRAN (ISLAMIC REPUBLIC OF)

## Abstract

Pain is one of the most prevalent and burdensome pediatric cancer symptoms for young children and their families. A significant proportion of pain episodes are experienced in environments where management options are limited, including at home. Digital innovations such as apps may have positive impacts on pain outcomes for young children in these environments. Our overall aim is to co-design such an app and the objective of this study was to explore the perceptions of children’s parents about app utility, needed system features, and challenges. We recruited parents of young children with cancer and multidisciplinary pediatric oncology clinicians from two pediatric cancer care centers to participate in audio-recorded, semi-structured, co-design interviews. We conducted interviews structured around technology acceptance and family caregiving theories until data saturation was reached. Audio-recordings were then transcribed, coded, and analyzed using thematic analysis. Forty-two participants took part in the process. Participants endorsed the concept of an app as a useful, safe, and convenient way to engage caregivers in managing their young child’s pain. Overall, the app was valued as a means to provide real-time, multimodal informational and procedural pain support to parents, while also reducing the emotional burden of pain care. Recommendations for intervention design included accessibility-focused features, comprehensive symptom tracking, and embedded scientific- and clinically-sound symptom assessments and management advice. Predicted challenges to app use included the workload burden it may place on parents and clinicians. The insights gathered will inform the design principles of our future childhood cancer pain digital research.

## Author summary

The lack of meaningful involvement of end-users in intervention development has been a key contributor to difficulties in effectively translating research findings into cancer practice and policy. There is risk that without the active engagement of children with cancer and their families in designing digital health innovations, researchers and clinicians will fall victim to an unfortunate cycle of producing underutilized evidence—resulting in poor adoption and a limited impact on patient outcomes. Pain is a particular problem for young children with cancer and real-time digital health interventions may be solutions for accessible, effective, and scalable cancer pain management. We are using an established user-centered co-design process to engage parents and pediatric oncology clinicians in the development of a cancer pain management app. Our work here summarizes the generative co-design phase of this process and the perceptions of parents and clinicians related to app usefulness and needed system features.

## Introduction

Pain is one of the most prevalent and burdensome pediatric cancer symptoms for children and their families despite the existence of evidence-based treatment guidelines [1,2]. The negative consequences of childhood cancer pain are many and include reduced child health-related quality of life, increased child and family distress, chronic pain in survivorship, and the potential for significant financial costs to healthcare systems and families [3–6]. Further, shifts to increasingly outpatient-based cancer care mean that children with cancer are experiencing pain in environments, such as home or school, where treatment options are limited [7].

Young children with cancer, including toddlers, preschoolers, and school-aged children, are particularly vulnerable to undermanaged pain due to their limited ability for pain self-report and their reliance on caregivers for pain management and treatment. Current research shows that digital innovations such as real-time smartphone-based pain management support apps may have positive impacts on pain outcomes in adolescents with cancer [5,8–10], but no investigations have been conducted into such tools for managing pain in younger children with cancer, especially outside the hospital setting. Recent reviews indicate that well-used digital patient or caregiver-focused cancer care interventions have potential for significantly positive health impacts—including around symptom distress [11,12]. Recommended features of digital cancer care apps, which may contribute to intervention utility and effectiveness, include cancer symptom tracking, reputable disease self-management training, social connectivity, and capacity for joint decision-making between patient/caregivers and clinicians [13–15].

To increase the likelihood of successful implementation of these digital health interventions within pediatric cancer care, it is vital that children, parents, other family caregivers and clinicians, are meaningfully integrated into the processes of intervention design, development, and evaluation [14]. Evidence shows that eliciting these perspectives supports the identification of barriers to intervention use, results in interventions with enhanced perceived effectiveness, activates a sense of ownership, and facilitates the successful delivery of digital health interventions [14,16–18].

The current study is part of a phased approach to the development and evaluation of a digital health app for the parent-led management of young children’s cancer pain. We focused on parents as app end-users because of the crucial role they play in pediatric cancer pain management [19–21]. Our overall co-design process utilized a Generative Co-Design Framework for Healthcare Innovation, which includes pre-design, co-design, and post-design phases—and which has been successfully used to develop pediatric health innovations [22]. This study describes the co-design step of innovation development as well as the post-design innovation requirements translation step. The co-design step builds upon foundational work understanding contextual circumstances surrounding a clinical issue and the lived experience of the issue—work that we have conducted to date [23]–to reveal the apparent and latent healthcare needs of participants. The requirements translation step involves utilizing co-design step themes to develop a set of intervention priority features and plans. Our future app development efforts will build on these results during parent-partnered software design feature sessions and the co-evaluation of a high-fidelity app prototype.

Given the significant issue of undermanaged pain in young children with cancer and the critical need to elicit user input early in the process of successful digital health intervention design, the objective of this study was to explore the perceptions of children’s parents and clinicians as they pertain to a parent-led real-time cancer pain management app, including possible utility, recommendations for needed system features, and potential challenges to implementation.

## Materials and methods

Our reporting is in accordance with the Consolidated Criteria for Reporting Qualitative Research (COREQ) [24] and Guidance for Reporting Involvement of Patients and the Public—Short Form (GRIPP2-SF) [25].

### Study approach, setting and participants

We used an inductive, qualitative descriptive approach for healthcare research [26], consistent with our goal to understand parent and clinician app perceptions and requirements. We recruited participants from the Hospital for Sick Children (SickKids) in Toronto, Ontario, Canada and the Children’s Hospital of Orange County (CHOC) in Orange, California, United States. We enrolled English-speaking parents who were the primary caregiver of a child (2–11 years) receiving treatment for any cancer diagnosis and who spent at least 25% of their cancer-care treatment time outside of the hospital and who had pain of any intensity in the preceding week. Child pain was determined by caregiver proxy-report. English-speaking multidisciplinary clinicians were included if they worked within the hematology/oncology program at either hospital and provided direct pain-related care to children spending at least 25% of their time during cancer treatment at home. We employed a purposive maximum variation sampling strategy with the aim of including parents who varied in age, sex, ethnicity, and their child’s diagnosis, and clinicians who varied by healthcare profession.

### Data collection

Following ethics board approval at the Hospital for Sick Children (#1000064500) and the University of California, Irvine (#190666), we obtained informed consent from participants. All participants completed demographic questionnaires and parents completed a Parental Pain Expression Perceptions (PPEP). The PPEP is a valid and reliable 9-item Likert scale-based questionnaire assessing parent knowledge and attitudes about pain expression in children, where higher scores represent greater misconceptions about pain expression [27].

Individual in-hospital, telephone, or online face-to-face semi-structured interviews were conducted by trained research team members (AC, HGP, LBT) with no previous relationship with participants. We used a combination of two behaviour change-related theoretical models to structure our interview guide. (**S1 Appendix**) First, the Unified Theory of Acceptance and Use of Technology 2 (UTAUT2) framework guided questions related to app aspects may improve acceptability and utility.[28] The use of the UTAUT framework to structure digital health intervention development has been suggested to support future cancer app implementation into clinical practice [14]. Van Houtven’s model of needed informal caregiver supports, an evidence-based theory describing the supports caregivers require to conduct caregiving activities, [29] allowed exploration of app features needed to improve app effectiveness. Similar interview guides were successfully used to co-design the currently developed Pain Squad+ pain management app for adolescents with cancer [30] and to study salient home-based pediatric cancer care issues [31]. We audio-recorded all interviews and handwritten field-notes were taken. We conducted interviews concurrently with our analyses until themes relevant to our study aim reached saturation.

### Data analysis

Interview audio-recordings were transcribed into electronic documents and uploaded to NVivo software version 11.4.0. Using an inductive approach, and to enhance trustworthiness [32], transcripts were coded in duplicate with reference to field-notes by four independent research assistants (SS, KH, TM, and MZ). Following the method of Braun and Clarke [33], we read through the dataset multiple times. As part of a researcher triangulation process [32], we further discussed dataset features as a group before creating several coding categories [32]. Coding proceeded using a statement-by-statement approach and codes were organized into themes and subthemes. The creation of themes was an iterative process where themes were continuously reviewed and compared against the narrative exemplars until a final thematic framework was established. The research team (SS, KH, TM, and LJ) met multiple times to ensure themes accurately reflected participant narratives through comparison to the raw data [32]. Consensus building exercises were used to refine themes and subthemes, where needed, and we kept an audit trail detailing decision making.

## Results

A total of 42 participants—21 parents and 21 clinicians—were interviewed between December 2019 and September 2020. All approached participants agreed to participate. The mean (SD) total interview times were 33 (15; range = 14–67) minutes and 33 (7; range = 22–45) minutes for parents and clinicians, respectively. Participant characteristics are shown in **Table 1**. Most parents were within the 30–39 years of age category (n = 13, 62%) and mothers (n = 17, 81%). The mean (SD) parent-reported PPEP scores were 33 (10), indicating moderately low child pain misconceptions. Clinicians were most often registered nurses (n = 9, 43%) and had a mean average 10 years of clinical experience.

**Table 1:** Participant characteristics.

Characteristic	n (%)	M (SD)
**Parents**		
Pain perception knowledge and attitudes (total PPEP^a^ score)		33 (10)
Caregiver type		
*Mother*	17 (81)	
*Father*	3 (14)	
*Stepmother*	1 (5)	
Marital status		
*Married*	19 (90)	
*Divorced*	1 (5)	
*Single*	1 (5)	
Sex		
*Female*	18 (86)	
*Male*	3 (14)	
Age range (years)		
*20–29*	3 (14)	
*30–39*	13 (62)	
*40–49*	5 (24)	
Parent-identified ethnicity		
*White*	8 (38)	
*Hispanic/Latinx*	5 (24)	
*Chinese*	2 (10)	
*South Asian*	2 (10)	
*Arab*	1 (5)	
*Armenian*	1 (5)	
*Black*	1 (5)	
*Korean*	1 (5)	
Highest level of education		
*College/University*	9 (43)	
*Professional School/Graduate Degree*	7 (33)	
*High School*	4 (19)	
*Teaching Credential*	1 (5)	
Primary language		
*English*	16 (76)	
*Arabic*	1 (5)	
*Cantonese*	1 (5)	
*Korean*	1 (5)	
*Tamil*	1 (5)	
*Urdu*	1 (5)	
Child age (years)		5.3 (2.7)
Child cancer diagnosis		
*Acute lymphoblastic leukemia*	9 (43)	
*Acute myeloid leukemia*	4 (19)	
*Langerhans cell histiocytosis*	2 (10)	
*Neuroblastoma*	2 (10)	
*Acute promyelocytic leukemia*	1 (5)	
*Lymphoma*	1 (5)	
*MYH9-related disorder*	1 (5)	
*Osteosarcoma*	1 (5)	
Time since child diagnosis (years)		0.9 (1.4)
**Clinicians**		
Profession		
*Advanced Practice Nurse*	5 (24)	
*Oncologist*	1 (5)	
*Physical Therapist*	3 (14)	
*Psychologist*	1 (5)	
*Registered Nurse*	9 (43)	
*Social Worker*	2 (10)	
Sex		
*Female*	21 (100)	
*Male*	0 (0)	
Age range (years)		
*20–29*	7 (33)	
*30–39*	8 (38)	
*40–49*	5 (24)	
*50–59*	1 (5)	
Highest level of education		
*Bachelor’s Degree*	7 (33)	
*Master’s Degree*	10 (48)	
*Doctoral Degree*	3 (14)	
*Medical Degree*	1 (5)	
Clinical work experience (years)		10(9)
Pediatric oncology clinical work experience (years)		8(8)

^a^ PPEP, **Parental Pain Expression Perceptions.**

We organized our data into four themes to describe parent and clinician perceptions of the utility of digital cancer pain intervention, recommendations for intervention components, and perceived challenges to app implementation.

### The need for and value of a digital cancer pain management intervention

#### Informational and procedural support for parents needed

Both parents and clinicians considered a pain management app to be a useful, safe, and convenient way to empower parental management of young children’s pain in the home environment.

“…Many times there are pain complaints that can be managed at home before you have to get to that threshold of coming in…” (Physical therapist, 6 years in pediatric oncology)

The app was expected to be especially useful to parents of children newly diagnosed with cancer. Several current deficiencies with at-home pain management for young children were also discussed. Informational and procedural support for parents was needed and valued. Both groups noted that parents already possessed a strong suite of child pain management skills, but a means to support the implementation of this knowledge would be useful.

“…If there’s a way of…teach[ing strategies and timing] to the parents and tell[ing] them that this is something that you could also do to help your son whenever he’s home or whenever he’s experiencing pain, that would be helpful.” (Mother of a 6-year-old)

“…I think sometimes when they go home, a thing they might struggle with is [deciding] when do I give this morphine? Am I giving too much? Am I giving too little?” (Nurse, 1.5 years in pediatric oncology)

#### Real-time interdisciplinary pain care needed

Clinicians described current clinical practices of learning about young children’s cancer pain as not comprehensive and often reliant on reviews of at-home medication use rather than lived experiences of pain. Procedural support to allow parents “to track when [their child is] having the pain and how long it’s been” (Nurse, 3 years in pediatric oncology) would provide in-depth information to healthcare teams and facilitate communication between parents and clinicians. Parents and clinicians also perceived pain to be better managed in hospital and described a need for connection to the interdisciplinary team and associated pain treatments at home.

“It’s nice to feel connected [to the team] when you’re at home.” (Mother of a 4-year-old)

“[The app should] offer ‘real time’ chat with a medical provider in case [parents] are not sure what to do specifically.” (Psychologist, 2 years in pediatric oncology)

#### Means to negotiate the emotional burden of pain care on parents needed

Parents alluded to the often-overwhelming task of managing pain at home stating, “You panic sometimes when your child is sick. You’re like, “What’s going on? How much [pain medication] do I give? What do I do?” (Mother of a 4-year-old). The numerous direct and indirect cancer care tasks for which parents assumed responsibility—such as medication administration and appointment tracking—amplified this strain and a tool that could offer parental emotional support was needed.

“Yeah, and I can’t apply that to her sometimes… like, I just can’t do it. You know she’s in pain or she’s really sick or she’s not in a good mood because of what’s, what’s happening and I just want something to help me get through this, you know?” (Mother, child age unknown)

“I think that it would be helpful because the parents are so overwhelmed and there’s so many things that they try and keep track of. There’s meds and chemotherapy and all that, so to have something dedicated to pain where it would also be easy to [track]… would be helpful to them” (Nurse, 3.5 years in pediatric oncology)

### Recommendations pertaining to pediatric digital health interventions in general

Parents and clinicians made recommendations that might be applied broadly to most parent-led digital health interventions, beyond only apps targeting pediatric cancer pain.

#### Usability-related recommendations: accessibility, tracking capacity, and gamification

Participants particularly noted the need to embed accessibility features in intervention design, emphasizing that these “app[s] should be made as easy as possible so that anybody can use” (Mother of a 4-year-old). To improve intervention access for parents, participants recommended app availability in multiple languages, the use of non-clinical lay terms for parents, a text-to-audio feature, and supplemental instructions that users could review as needed.

“…We have a lot of patients that have come from different countries, and I guess it would be difficult for some parents to use an app, especially if it’s only provided in English…”(Nurse, 1.5 years in pediatric oncology)

“A majority of our population isn’t English-speaking so it would be a barrier [if app only available in English]” (Nurse, 9.5 years in pediatric oncology)

Participants recommended integrating a calendar feature to act as a centralized hub with scheduled medication and medical appointment reminders, and to allow for child symptom tracking. Many participants also suggested adding gamified elements such as avatars and pictorial representations.

“Yeah, [gamified] challenges. I love them *laughs* and it makes you, you know, want to [engage] more…” (Mother, child age unknown)

Such gamification was considered to encourage family-oriented digital care by making apps child-appropriate and encouraging child engagement in app-based care. Specific avatar recommendations were to include features reflective of the parent and child (e.g., diverse skin tones, range of heights and weights, features such as glasses and prosthetics).

#### Credibility-related recommendations: clinician integration, quality evidence base, personalization, and clinical disclaimer

Clinician integration within digital health interventions was frequently cited as important.

“I like the idea that…the nurse can contact [you] when it’s for the extreme pain. It’s a nice feature.” (Mother of a 4-year-old)

Considering this integration, participants recommended a software-embedded threshold for clinical follow-up that, if surpassed, would result in notification to the healthcare team. Such notifications were suggested to ensure child safety and to build technologies that enhance, rather than replace, care delivered by clinicians. This sharing of app-generated health reports with clinicians was considered an important means to support family-centered care by “sort of opening a door to a conversation that maybe [parents and clinicians] wouldn’t have had” (Oncologist, 1 year in pediatric oncology). To enhance credibility, participants strongly recommended app information be clinically-sound, up-to-date, research-based, and personalized to users, including by implementing software algorithm-based personalization to the child. Finally, clear descriptions of the limits of app-based clinical support should be embedded.

“Pain is subjective so it’s hard to know what they’re going to do with that but I think there should always be a disclaimer: “If you feel that you know [what to do], you could try this, but, if you feel the pain is escalating please call 9-1-1 or please take your child to the nearest emergency room” (Social worker, 25 years in pediatric oncology)

Participants highlighted that such disclaimers would build trust with the intervention.

### Recommendations pertaining to parent-led digital cancer pain management specifically

#### Detailed patient history

Participants suggested including a feature that enabled the storage of a child’s pain history, medical history, current medications, allergies, social history, and family history. This information could be updated by both parents and clinicians and would support personalizing pain management advice to a child.

“Weight, height…I would get that down…and then the feeding and pain management or the pain history.” (Mother of a 2-year-old)

“Things that would cause pain, like their diagnosis, recent surgery, or chronic history of pain.” (Nurse, 12 years in pediatric oncology)

“It would be helpful to know the symptoms. A lot of times when a parent comes in, they don’t have symptoms recorded… Sometimes they’ll say “Yeah, [my child has] pain”—sometimes we don’t know. It’s kind of vague when they tell us about when they’re feeling the pain or describe the pain. Is it achy? Is it sharp? Is it shooting?” (Nurse, 1.5 years in pediatric oncology)

#### Comprehensive pain assessments

Both parents and clinicians strongly recommended that multidimensional pain assessment tools be embedded in the app. These tools should measure pain as a sensory phenomenon (e.g., pain frequency, duration, severity, and cause), as well as consider the impact of pain on a child’s affective state and a child’s activities of daily living, including ability to take medications.

“I think it would be helpful to track…when [the child] had treatment and associated pain. That could be helpful for collecting data about when these children are experiencing pain. That may be helpful also for researchers to link the effects of pain management and help people better understand the real side effects of [cancer treatment].” (Mother of a 5-year-old)

App-embedded pain assessments should be valid and implemented the moment pain occurs for the sake of accuracy. Assessments should also be appropriate to the age, developmental stage, and should be repeated once a pain management intervention was implemented. Assessments should consider the abilities of the younger child with cancer, recognizing that parent proxy assessments may be required for children unable to self-report their pain.

“It’s really, really hard for young kids, but I think giving more adjectives that parents could use or other ways to describe different types of pain” (Physical therapist, 6 years in pediatric oncology)

Parents also suggested building in capacity to record other aspects of their child’s physical status that may be related to pain (e.g., bowel movement patterns, nausea and vomiting). Parents felt confident in their abilities to understand and implement app-based pain management instructions, noting familiarity with many the mobile apps, including health apps, that they currently use.

#### Multimodal integrative pain management advice

Parents required physical, psychological, and pharmacological cancer pain management advice. App-integrated physical advice included deep breathing or gentle exercise and stretching. Recommended psychological advice was distraction including playing, relaxation, and massage, and advice to emotionally support parents in their role as pain caregivers.

“Distraction things first. Like massage, hot packs, cold packs, and stuff like that.” (Mother, child age unknown)

“…there’s other pain management strategies that they can use at home that we don’t use that much in the hospital, like relaxation and distraction and all that kind of stuff…” (Nurse, 1.5 years in pediatric oncology)

Embedding information on how to manage medication side effects was also suggested.

#### Capacity to track pain overtime

Both parents and clinicians expressed a need for a digital intervention to track child pain overtime and provide visual data representations. This was considered to enable pain pattern recognition and to support family-clinician conversations about pain.

“Yeah, to be able to track daily and have a place that we can consolidate that data ultimately.” (Father of 6-year-old)

Within the app, parents and clinicians also recommended tracking which pain management strategies were used, and the effectiveness of each strategy.

“I think it would be useful as a tracking method because… there’s so many meds, so many chemos, so many appointments, that you kind of get lost in all of it. If there was ever a time that they need to come to [the emergency department] it would just be a really easy way to be like, “This is when my pain started. This is what they did.” (Nurse, 3.5 years in pediatric oncology)

### Perceived challenges associated with implementing a digital pain management intervention

#### Threshold-setting around clinician-based support

Most prominently, participants discussed potential difficulty in setting a software-embedded pain threshold beyond which healthcare provider support would be initiated. Participants specifically discussed the subjective nature of pain and a resultant need to individualize pain thresholds to each child and their family context.

“[When should doctors intervene in parent pain management at home?] Sometimes, but it just depends. Depends on [the] situation, okay?” (Mother, child age unknown)

“I think that’s what’s hard, because pain is very subjective, and…so I think that’s where it’s hard to pinpoint [when the healthcare team should be alerted].” (Advanced practice nurse, 12 years in pediatric oncology)

#### Introduction of a new burden on parents

Clinicians discussed the potential for the app to add burden for parents who are already strained with caregiving tasks for their child.

“But if they never heard of that technique before…are they going to be wasting time thinking, ‘…What’s that? What does that mean? How do you do that breathing? What does relaxation mean?’ Like, right? Is that going to add more stress?” (Social worker, 25 years in pediatric oncology)

However, several parents considered use of the app, including once or twice a day, to be feasible.

#### Difficultly in integrating into clinician workflows

A potential challenge related to how to successfully integrate app-based data monitoring and pain support into daily clinician workflows was discussed.

“And I can’t imagine anyone here has an easy or laid-back position. I imagine everybody’s role here is pretty impactful and a lot of you are busy most of the time so…” (Mother of a 2-year-old)

“I think in an ideal world, that’d be a great idea but I just don’t know who would take on that responsibility. The best person I can think of would be [the family’s primary] nurse but I also know that they’re so busy with other things.” (Nurse, 3 years in pediatric oncology)

Participants highlighted that successfully integrating the app into parent daily schedules and clinician workflows would depend on demonstrating positive impacts on child pain outcomes that outweigh any burden of use.

#### Potential to result in family hyperawareness of and sensitization to symptoms

Participants also worried that frequent pain monitoring may lead parents and children to become acutely aware and subsequently hypersensitized to pain.

“I’ve experienced some parents being hyper vigilant.” (Social worker, 25 years in pediatric oncology)

## Discussion

This study investigated the perspectives of parents of young children with cancer and pediatric cancer care clinicians as they pertain to the design and delivery of digital health interventions targeting children’s pain. Based on the UTAUT2 constructs and van Houtven’s framework for family caregiver engagement, an app-based solution appears as an acceptable support for parents, especially considering the limited at-home family-led pain management strategies and the often-overwhelming nature of managing pain independently in care settings other than the hospital. Parents and clinicians recommended ways to improve the utility of such an intervention, including by integrating accessibility features and ensuring high-quality pain management content. Participants recommended needed digital features including embedded multidimensional pain assessments, multi-modal pain management support, and pain tracking over time. Critical to the app was the inclusion of detailed child and family information to support the personalization of provided pain support. These findings contribute to a growing literature on the support requirements of parents caring for a child with cancer [34–36]. Based on our findings, we have created a list of app design features (**Table 2**). This feature list resembles that which we have previously vetted with parents through a low-fidelity app usability testing exercise [21].

**Table 2:** Potential design features of a parent-led childhood cancer pain management app.

Anticipated design feature	Associated theme(s) and subtheme(s)
Daily multidimensional child pain assessment	• Recommendations pertaining to parent-led digital cancer pain management specifically > Comprehensive pain assessments
Evidence-based care cards explicitly directing parent pain management activities	• The need for and value of a digital cancer pain management intervention > Informational and procedural support for parents needed • Recommendations pertaining to pediatric digital health interventions in general > Credibility-related recommendations: quality evidence base • Recommendations pertaining to parent-led digital cancer pain management specifically > Multimodal integrative pain management advice
Nurse chat feature enabling clinician contact for parents	• The need for and value of a digital cancer pain management intervention > Real-time interdisciplinary pain care needed • Recommendations pertaining to pediatric digital health interventions in general > Credibility-related recommendations: clinician integration
Caregiver resources with information-based psychosocial support for parents	• The need for and value of a digital cancer pain management intervention > Means to negotiate the emotional burden of pain care on parents needed
Reviewable pain report trends	• Recommendations pertaining to pediatric digital health interventions in general > Usability-related recommendations: tracking capacity • Recommendations pertaining to parent-led digital cancer pain management specifically > Capacity to track pain overtime
Parent and child profile personalizing app	• Recommendations pertaining to pediatric digital health interventions in general > Credibility-related recommendations: personalization • Recommendations pertaining to parent-led digital cancer pain management specifically > Detailed patient history • Recommendations pertaining to pediatric digital health interventions in general > Usability-related recommendations: accessibility
Clinical care disclaimer	• Recommendations pertaining to pediatric digital health interventions in general > Credibility-related recommendations: clinical disclaimer
Integrated behaviour change techniques such as motivational notifications, badges	• Recommendations pertaining to pediatric digital health interventions in general > Usability-related recommendations: gamification

Existing literature highlights the importance of involving end-users in the design and evaluation of apps for specific health conditions, particularly noting the positive impact on intervention uptake into cancer care [14] and the achievement of an intended health goal [37]. Meaningful user engagement also enables understanding of the key design features that will facilitate successful digital interactions, as well as features that may limit use (e.g., poor integration with daily workflows) [37–39].

Recommendations for accessible language features, the use of lay terms and supplemental health information, and the use of gamification to engage users are supported by the existing literature [40,41]. Research shows that language barriers in healthcare lead to miscommunication between medical professionals and patients and the implementation of language accessibility features improves the quality of healthcare delivery and patient safety [42]. App gamification also improves the user experience, app accessibility, app appeal, and engagement [43].

Our results show the critical importance of providing high-quality evidence-based health advice to app users, which aligns with a digital health needs assessment conducted with the parents of children with cancer [44]. This also reflects the findings of a recent systematic review that identified standards for mobile health apps, including that they should name content authors and their professional qualifications and utilize scientific evidence as the basis for quality content [45]. Our participants also recommended the implementation of app health disclaimers, aligning with current health app guidance to disclose possible risks to users and include warnings that apps are not intended to replace health professional care [45]. Parents in our study particularly emphasized their preference that the app support direct communication with their child’s clinical team—a finding that is consistent across studies within [13,44] and outside of [46] digital cancer care. Attention must be paid to the content and frequency of app-mediated communications between clinicians and families and these factors considered in app quality assurance protocols [34] and clinical staffing profiles [46].

The need to embed a comprehensive multidimensional pain assessment tool and multi-modal integrative pain management advice in the app reflects current considerations for managing cancer pain. This recommendation reflects a clinical need to implement a biopsychosocial approach to pain assessment and management, including treating childhood cancer pain with evidence-based pharmacological, psychological, physical, and complementary and alternative medicine techniques [1]. Consideration of the time to complete comprehensive assessments is also needed as previous research in pediatric cancer pain apps has shown lengthy questionnaires to be a drawback to app use [18].

Predicted challenges associated with digital health interventions include the potential for the app to burden parents and clinicians. Studies of high burden apps often include high participant attrition, which is rooted in the fundamental challenges of keeping participants engaged in intervention use over time [47]. Further, the work of deploying digital health tools within routine clinical workflows is notoriously challenging [46] and requires carefully conceptualized and contextualized action plans [48]. Difficulties in setting app-based pain thresholds for clinician-driven pain management support will require a personalized approach to pain care and the ability to tailor co-designed digital interventions to the needs of unique users and contexts.

Our findings support those from separate UTAUT2-directed research, which suggest that additional constructs may be required within the theoretical model. New evidence shows that user acceptance of health apps is influenced by user trust in app data security and quality, as well as perceived risks associated with a health condition [49]. In this study, participants highlighted the need for high-quality cancer pain evidence and clinician support within the app and noted the negative impacts of child pain as an impetus for app use.

Strengths of our study include the integration of multiple perspectives and our collection of data from two international pediatric cancer care centers. Limitations include potential social desirability response bias whereby parents and clinicians may have withheld negative comments during interviews with our team. However, participants were informed that all feedback would be valued equally. Additionally, the mode by which we conducted interviews was not standardized and we instead held interviews via telephone, online, and in-person. However, research shows good comparability between the content and depth of interviews conducted in-person and otherwise [50,51]. The generalizability of our results is limited by our inability to recruit many fathers or male clinicians to the study. We also did not include children’s perspectives in this co-design process. Engaging with younger children using established methodologies [52] is a goal of our future research. Finally, we did not conduct participant checking of either the transcriptions or final thematic analysis.

This study provides recommendations from parents and clinicians on the design and delivery of digital pediatric cancer pain interventions. These recommendations can be readily used by parents, clinicians, engineers, and researchers participating in intervention development. Notably, several recommendations are directly applicable to the design of many pediatric digital health interventions—beyond those specific to childhood cancer pain. We recommend that future investigators who endeavor to develop health apps implement a co-design approach within their work. Such end-user participation can reveal critical intervention design requirements that may boost the relevance of app content and clinical effectiveness. The recommendations gathered in the present study will continue to inform the design principles of our future childhood cancer pain.

## Supporting information

S1 Appendix Semi-structured interview guide for parents and clinicians. (DOCX)

## References

[1] Loeffen EAH, Mulder RL, Font-Gonzalez A, Leroy PLJM, Dick BD, Taddio A, et al. Reducing pain and distress related to needle procedures in children with cancer: A clinical practice guideline. Eur J Cancer. 2020;131: 53–67. doi: 10.1016/j.ejca.2020.02.039 32302949

[2] Loeffen EAH, Kremer LCM, Wetering MD van de, Mulder RL, Font-Gonzalez A, Dupuis LL, et al. Reducing pain in children with cancer: Methodology for the development of a clinical practice guideline. Pediatr Blood Cancer. 2019;46: e27698. doi: 10.1002/pbc.27698 30848078 PMC9286396

[3] Schulte FSM, Patton M, Alberts NM, Kunin-Batson A, Olson-Bullis BA, Forbes C, et al. Pain in long-term survivors of childhood cancer: A systematic review of the current state of knowledge and a call to action from the Children’s Oncology Group. Cancer. 2021;127: 35–44. doi: 10.1002/cncr.33289 33112416 PMC7875461

[4] Tutelman PR, Chambers CT, Urquhart R, Fernandez CV, Heathcote LC, Noel M, et al. When “a headache is not just a headache”: A qualitative examination of parent and child experiences of pain after childhood cancer. Psychooncology. 2019;28: 1901–1909. doi: 10.1002/pon.5170 31276614

[5] Stinson JN, Jibb LA, Nguyen C, Nathan PC, Maloney AM, Dupuis LL, et al. Construct validity and reliability of a real-time multidimensional smartphone app to assess pain in children and adolescents with cancer. Pain. 2015;156: 2607–2615. doi: 10.1097/j.pain.0000000000000385 26580680

[6] Barbera L, Taylor C, Dudgeon D. Why do patients with cancer visit the emergency department near the end of life? CMAJ. 2010;182: 563–568. doi: 10.1503/cmaj.091187 20231340 PMC2845683

[7] Fortier MA, Wahi A, Bruce C, Maurer EL, Stevenson R. Pain management at home in children with cancer: a daily diary study. Pediatr Blood Cancer. 2014;61: 1029–1033. doi: 10.1002/pbc.24907 24376073

[8] Jibb LA, Stevens BJ, Nathan PC, Seto E, Cafazzo JA, Johnston DL, et al. Implementation and preliminary effectiveness of a real-time pain management smartphone app for adolescents with cancer: a multicenter pilot clinical study. Pediatr Blood Cancer. 2017;64: e26554. doi: 10.1002/pbc.26554 28423223

[9] Tiozzo E, Fondi S, Biagioli V, Piccinelli E, Alibrandi F, Gawronski O, et al. electronic assessment and tracking of pain at home: a prospective study in children with hematologic or solid tumors. J Pediatr Oncol Nurs. 2021;38: 82–93. doi: 10.1177/1043454220975443 33269620

[10] Fortier MA, Chung WW, Martinez A, Gago-Masague S, Sender L. Pain buddy: A novel use of m-health in the management of children’s cancer pain. Comput Biol Med. 2016;76: 202–214. doi: 10.1016/j.compbiomed.2016.07.012 27479493 PMC5639256

[11] Marzorati C, Renzi C, Russell-Edu SW, Pravettoni G. telemedicine use among caregivers of cancer patients: systematic review. J Med Internet Res. 2018;20: e223. doi: 10.2196/jmir.9812 29914858 PMC6028768

[12] Adriaans DJ, Daele ATD, Bakel MJHM van, Nieuwenhuijzen GA, Teijink JA, Heesakkers FF, et al. Digital self-management support tools in the care plan of patients with cancer: review of randomized controlled trials. J Med Internet Res. 2021;23: e20861. doi: 10.2196/20861 34184997 PMC8278296

[13] Vaffis S, Whaley S, Axon DR, Hall-Lipsy E, Hincapie A, Slack M, et al. Features of cancer mhealth apps and evidence for patient preferences: scoping literature review. JMIR Cancer. 2023;9: e37330. doi: 10.2196/37330 37115587 PMC10182455

[14] Ardito V, Golubev G, Ciani O, Tarricone R. Evaluating barriers and facilitators to the uptake of mHealth apps in cancer care using the consolidated framework for implementation research: scoping literature review. JMIR Cancer. 2023;9: e42092. doi: 10.2196/42092 36995750 PMC10131717

[15] Charbonneau DH, Hightower S, Katz A, Zhang K, Abrams J, Senft N, et al. Smartphone apps for cancer: a content analysis of the digital health marketplace. Digit Heal. 2020;6: 2055207620905413. doi: 10.1177/2055207620905413 32110428 PMC7016299

[16] Greenhalgh T, Hinton L, Finlay T, Macfarlane A, Fahy N, Clyde B, et al. Frameworks for supporting patient and public involvement in research: Systematic review and co-design pilot. Health Expect 2019;22: 785–801. doi: 10.1111/hex.12888 31012259 PMC6737756

[17] Teychenne M, Apostolopoulos M, Ball K, Olander EK, Opie RS, Rosenbaum S, et al. Key stakeholder perspectives on the development and real-world implementation of a home-based physical activity program for mothers at risk of postnatal depression: a qualitative study. BMC Public Health. 2021;21: 361. doi: 10.1186/s12889-021-10394-8 33593324 PMC7885569

[18] Jibb LA, Stevens BJ, Nathan PC, Seto E, Cafazzo JA, Johnston DL, et al. Perceptions of adolescents with cancer related to a pain management app and its evaluation: qualitative study nested within a multicenter pilot feasibility study. JMIR mHealth uHealth. 2018;6: e80. doi: 10.2196/mhealth.9319 29625951 PMC5910537

[19] Twycross A, Parker R, Williams A, Gibson F. Cancer-Related pain and pain management: sources, prevalence, and the experiences of children and parents. J Pediatr Oncol Nurs. 2015;32: 369–384. doi: 10.1177/1043454214563751 25736032

[20] Tutelman PR, Chambers CT, Stinson JN, Parker JA, Fernandez CV, Witteman HO, et al. Pain in children with cancer. Clin J Pain. 2018;34: 198–206. doi: 10.1097/ajp.0000000000000531 28678061

[21] Jibb LA, Liu W, Stinson JN, Nathan PC, Chartrand J, Alberts NM, et al. Supporting parent capacity to manage pain in young children with cancer at home: co-design and usability testing of the PainCaRe app. Paediatr Neonatal Pain. 2023. doi: 10.1002/pne2.12097 PMC11514300 39473834

[22] Bird M, McGillion M, Chambers EM, Dix J, Fajardo CJ, Gilmour M, et al. A generative co-design framework for healthcare innovation: development and application of an end-user engagement framework. Res Involv Engagem. 2021;7: 12. doi: 10.1186/s40900-021-00252-7 33648588 PMC7923456

[23] Jibb LA, Stinson JN, Nathan PC, Chartrand J, Alberts NM, HIldenbrand A, et al. “Just ease the stress of it”: perspectives and intervention suggestions from parents managing cancer pain in young children at home. Pediatr Blood Cancer. 2021;68: S50–S51. doi: 10.1002/pbc.29349

[24] Tong A, Sainsbury P, Craig J. Consolidated criteria for reporting qualitative research (COREQ): a 32-item checklist for interviews and focus groups. Int J Qual Health Care. 2007;19: 349–357. doi: 10.1093/intqhc/mzm042 17872937

[25] Staniszewska S, Brett J, Simera I, Seers K, Mockford C, Goodlad S, et al. GRIPP2 reporting checklists: tools to improve reporting of patient and public involvement in research. BMJ. 2017;358: j3453. doi: 10.1136/bmj.j3453 28768629 PMC5539518

[26] Bradshaw C, Atkinson S, Doody O. Employing a qualitative description approach in health care research. Global Qual Nurs Res. 2017;4: 2333393617742282. doi: 10.1177/2333393617742282 29204457 PMC5703087

[27] Zisk RY, Grey M, MacLaren JE, Kain ZN. Exploring sociodemographic and personality characteristic predictors of parental pain perceptions. Anesth Analg. 2007;104: 790–798. doi: 10.1213/01.ane.0000257927.35206.c1 17377084

[28] Venkatesh Thong, Xu. Consumer acceptance and use of information technology: extending the unified theory of acceptance and use of technology. MIS Q. 2012;36: 157. doi: 10.2307/41410412

[29] vanHoutven CH, Voils CI, Weinberger M. An organizing framework for informal caregiver interventions: detailing caregiving activities and caregiver and care recipient outcomes to optimize evaluation efforts. BMC Geriatr. 2011;11: 77. doi: 10.1186/1471-2318-11-77 22107600 PMC3258201

[30] Jibb LA, Stevens BJ, Nathan PC, Seto E, Cafazzo JA, Stinson JN. A smartphone-based pain management app for adolescents with cancer: establishing system requirements and a pain care algorithm based on literature review, interviews, and consensus. JMIR Res Protoc. 2014;3: e15. doi: 10.2196/resprot.3041 24646454 PMC3978558

[31] Jibb LA, Chartrand J, Masama T, Johnston DL. Home-based pediatric cancer care: perspectives and improvement suggestions from children, family caregivers, and clinicians. JCO Oncol Pract. 2021;17: e827–e839. doi: 10.1200/OP.20.00958 33914620

[32] Nowell LS, Norris JM, White DE, Moules NJ. Thematic analysis: striving to meet the trustworthiness criteria. Int J Qual Methods. 2017;16: 160940691773384. doi: 10.1177/1609406917733847

[33] Braun V, Clarke V. Using thematic analysis in psychology. Qual Res Psychol. 2006;3: 77–101. doi: 10.1191/1478088706qp063oa

[34] Roberts AE, Davenport TA, Wong T, Moon H-W, Hickie IB, LaMonica HM. Evaluating the quality and safety of health-related apps and e-tools: adapting the Mobile App Rating Scale and developing a quality assurance protocol. Internet Interv. 2021;24: 100379. doi: 10.1016/j.invent.2021.100379 33777705 PMC7985461

[35] Tan R, Koh S, Wong ME, Rui M, Shorey S. Caregiver stress, coping strategies, and support needs of mothers caring for their children who are undergoing active cancer treatments. Clin Nurs Res. 2020;29: 460–468. doi: 10.1177/1054773819888099 31709812

[36] Nieto BB, Muñoz CP, López MÁV. Needs assessment in parents of children affected by cancer: a qualitative perspective. Children. 2022;9: 1957. doi: 10.3390/children9121957 36553400 PMC9776725

[37] Powell L, Joddrell P, Parker J. Involving users in the evaluation of apps for specific health conditions. Stud Health Technol. 2017;242: 646–653. 28873866

[38] Floch J, Zettl A, Fricke L, Weisser T, Grut L, Vilarinho T, et al. User needs in the development of a health app ecosystem for self-management of cystic fibrosis: user-centered development approach. JMIR mHealth uHealth. 2018;6: e113. doi: 10.2196/mhealth.8236 29739742 PMC5964302

[39] Rodriguez DV, Lawrence K, Luu S, Yu JL, Feldthouse DM, Gonzalez J, et al. Development of a computer-aided text message platform for user engagement with a digital diabetes prevention program: a case study. J Am Med Informa Assoc. 2021;29: 155–162. doi: 10.1093/jamia/ocab206 34664647 PMC8714274

[40] Muñoz AO, Camacho E, Torous J. Marketplace and literature review of Spanish language mental health apps. Frontiers Digital Heal. 2021;3: 615366. doi: 10.3389/fdgth.2021.615366 34713093 PMC8521936

[41] Fijačko N, Gosak L, Cilar L, Novšak A, Creber RM, Skok P, et al. The effects of gamification and oral self-care on oral hygiene in children: systematic search in app stores and evaluation of apps. JMIR mHealth uHealth. 2020;8: e16365. doi: 10.2196/16365 32673235 PMC7381071

[42] Gerchow L, Burka LR, Miner S, Squires A. Language barriers between nurses and patients: a scoping review. Patient Educ Couns. 2020;104: 534–553. doi: 10.1016/j.pec.2020.09.017 32994104 PMC8011998

[43] Johnson D, Deterding S, Kuhn K-A, Staneva A, Stoyanov S, Hides L. Gamification for health and wellbeing: a systematic review of the literature. Internet Interv. 2016;6: 89–106. doi: 10.1016/j.invent.2016.10.002 30135818 PMC6096297

[44] Mueller EL, Cochrane AR, Bennett WE, Carroll AE. A survey of mobile technology usage and desires by caregivers of children with cancer. Pediatr Blood Cancer. 2018;15: e27359. doi: 10.1002/pbc.27359 30015371

[45] Llorens-Vernet P, Miró J. Standards for mobile health–related apps: systematic review and development of a guide. JMIR mHealth uHealth. 2020;8: e13057. doi: 10.2196/13057 32130169 PMC7078629

[46] Lazarevic N, Pizzuti C, Rosic G, Bœhm C, Williams K, Caillaud C. A mixed-methods study exploring women’s perceptions and recommendations for a pregnancy app with monitoring tools. npj Digit Med. 2023;6: 50. doi: 10.1038/s41746-023-00792-0 36964179 PMC10036977

[47] Amagai S, Pila S, Kaat AJ, Nowinski CJ, Gershon RC. Challenges in participant engagement and retention using mobile health apps: literature review. J Med Internet Res. 2022;24: e35120. doi: 10.2196/35120 35471414 PMC9092233

[48] Marwaha JS, Landman AB, Brat GA, Dunn T, Gordon WJ. Deploying digital health tools within large, complex health systems: key considerations for adoption and implementation. npj Digit Med. 2022;5: 13. doi: 10.1038/s41746-022-00557-1 35087160 PMC8795422

[49] Schretzlmaier P, Hecker A, Ammenwerth E. Suitability of the Unified Theory of Acceptance and Use of Technology 2 Model for predicting mhealth acceptance using diabetes as an example: qualitative methods triangulation study. JMIR Hum Factors. 2022;9: e34918. doi: 10.2196/34918 35262493 PMC8943545

[50] Woodyatt CR, Finneran CA, Stephenson R. In-person versus online focus group discussions: a comparative analysis of data quality. Qual Health Res. 2016;26: 741–749. doi: 10.1177/1049732316631510 26935719

[51] Sturges JE, Hanrahan KJ. comparing telephone and face-to-face qualitative interviewing: a research note. Qual Res. 2004;4: 107–118. doi: 10.1177/1468794104041110

[52] Youth-centred digital health interventions: a framework for planning, developing and implementing solutions with and for young people. Geneva: World Health Organization; 2021 July 19. Available from: https://www.who.int/publications/i/item/9789240011717.

