# Supplementary material for: Parent and clinician perceptions and recommendations on a pediatric cancer pain management app: A qualitative co-design study
Source: PLOS Digit Health. 2023 Nov 29;2(11):e0000169. doi: 10.1371/journal.pdig.0000169 (PMC10686487; doi:10.1371/journal.pdig.0000169)
Supplement: S1 Appendix — (DOCX) [file pdig.0000169.s001.docx]

**PROTOCOL NAME: Building mHealth Capacity to Manage Pain in Young Children with Cancer: Needs Assessment and Usability Testing for a Caregiver-led Pain Management App**

**PHASE 1: Caregiver interview guide**

**Why is this study being done?**

This study is being done because we would like to refine an app we have already made for children with cancer aged 12-18 and make it useful for children with cancer below the age of 12 and their caregivers.

The app is called Pain Squad+ and was developed by Dr. Jibb. Pain Squad+ helps kids with cancer to manage their pain using game-like themes. Kids can complete a pain questionnaire each morning and evening and at any other time they are in pain. Using the app kids see what different types of pain they feel, what makes the pain better or worse, how it affects daily activities and what can be done to deal with the pain. The app is a helpful way for kids and their caregivers to understand their pain better. It is also a good way to share information with treating doctors so they can help manage the child’s pain better. Studies done using this app show that it is practical tool that is useful and easy to use.

For this study, we will be talking to parents or main caregivers of children with cancer and asking them how this app can be made better. We will also be talking to health care staff who provide care to children with cancer. We will then make changes to the app based on what parents and health care staff tell us, so the app is as useful as possible to parents.

**Audio recording**

The interview will audio recorded. This is being done to make sure we document your responses as accurately as possible. If you do not want to be audio recorded, you cannot be part of this study. The tape will be typed out word for word (transcribed). After the typed reports (transcripts) have been verified, the tapes will be destroyed.

**Interview preamble**

“Hello. I am part of a research team that is working on building an app to support caregivers to manage their child’s cancer pain when they are at home. We have already developed an app like this for teens with cancer and we would like to make changes to it so caregivers can use it.

Here are some screenshots of the app for teens [show screenshots]. The app currently works by alerting teens twice daily to complete a short pain report. Teens can also complete pain reports any other time during the day if they would like. Whenever a teen reports that they are having pain, the app provides the teen with pain management advice such as reminders to take pain medications or suggestions to do things like relaxation. If a teen reports 3 pain times in a row where pain is rated as severe, an email is sent to a nurse at the hospital who can then call the teen and help with managing the pain.

If it is ok, now I would like to ask some questions about pain your child has had and if a pain app might help to manage that pain. Please remember that you do not have to answer any of the questions you are not comfortable with. You can also withdraw from the study at any time. If you withdraw, any data we

***UTAUT2 performance expectancy*** *(“Degree to which using a technology will provide benefits to consumers in performing certain activities”) and* ***van Houtven caregiver clinical knowledge*** *(“Required skills and knowledge to carry out caregiving activities”)*

1. What apps do you currently use?
   1. What is it about these apps that you like?
   2. How could these apps be improved?
2. Do you currently use any health-related apps like Fitbit, Apple Health, or others?
3. What health apps do you use?
4. Why do you use them?
5. What are your general thoughts related to an app like this?
6. What do you like best about the idea of this app?
7. What do you like least about the idea of this app?
8. Do you think this app would be a useful tool for you to record and manage your child’s pain?
   1. Why or why not?

***UTAUT2 effort expectancy*** *(“Degree of ease associated with consumers’ use of technology”) and* ***van Houtven quantity of caregiving*** *(“Objective burden, including the time spent caregiving”)*

1. Do you predict any difficulties with using the app?
2. Technical problems with your phone?
3. Not knowing how to use the app?
4. Not understanding the text in the app?
5. Not knowing when to use the app with your child?
6. How confident are you that you would be able to use an app to record your child’s pain?
7. What would make you more or less confident?

***UTAUT2 social influence*** *(“Extent to which consumers perceive that important others believe they should use a particular technology”) and* ***van Houtven support seeking skills*** *(“developing organizational, tactical, and recruiting skills that help support caregiving efforts”)*

1. When should your child’s doctors and nurses help you manage your child’s pain using the app?
   1. Can you make pain treatment choices with the app by yourself?
   2. What are your thoughts about having a nurse see how you recorded your child’s pain on the app from the hospital and then call you at home to help with pain management?
   3. How bad should the pain before your child’s doctors and nurses have to know about it?

***UTAUT2 facilitating conditions*** *(“consumers’ perceptions of the resources and support available to perform a behavior”) and* ***van Houtven psychological skills and resources*** *(“coping and self-efficacy enhancing skills for caregivers”)*

1. Now that you have an idea of the app we want to build, do you have any suggestions for the app?
2. What questions do you think the app should ask you to answer when you record your child’s pain?
3. How bad the pain is?
4. How much the pain is getting in the way of your child doing things like eating or taking medications?
5. How much the pain is getting in the way of your child playing?
6. How much the pain is getting in the way of your child sleeping?
7. Where the pain is happening on your child’s body?
8. How much the pain is bothering your child?
9. Anything else?
10. What sort of pain management help should the app give you so that you can take care of your child’s pain?
11. Should the app help you make choices about when to give pain medications? Can you tell me more about that?
12. Should the app help you make choices about when to doing physical things with your child like exercising to manage pain? Can you tell me more about that?
13. Should the app help you make choices about helping your child do things like relaxing, mediating and distraction to manage pain? Can you tell me more about that?
14. If this app could give you pain management advice when your child had pain, how confident are you that you could do what the app recommended?
15. What would make you more or less confident?

***UTAUT2 hedonistic motivation*** *(“The fun or pleasure derived from using a technology”)*

1. Do you have any ideas about what the app should look like?
   1. Colors? Text? Pictures?
2. What types of things would the app have to have for you to want to use it?
   1. Alarms to remind you to record your child’s pain?
   2. Gamification? This means things like app points or awards you would get for using the app

***UTAUT2 habit*** *(“The extent to which people tend to perform behaviors automatically because of learning”)*

1. How many times a day should you use the app to record your child’s pain?
2. Do you think using the app once or twice a day is a realistic thing to do? Can you tell me more about that?

**Closing question**

1. Is there anything else you would like to mention about cancer pain or the app?

**Interview conclusion**

“Thank you very much for participating in this study. The information you have provided is invaluable to use as well refine and develop an app for parents providing pain management to their young children with cancer.”

**PROTOCOL NAME: Building mHealth Capacity to Manage Pain in Young Children with Cancer: Needs Assessment and Usability Testing for a Caregiver-led Pain Management App**

**Clinician interview guide**

**Why is this study being done?**

This study is being done because we would like to refine an app we have already made for children with cancer aged 12-18 and make it useful for children with cancer below the age of 12 and their caregivers.

The app is called Pain Squad+ and was developed by Dr. Jibb. Pain Squad+ helps kids with cancer to manage their pain using game-like themes. Kids can complete a pain questionnaire each morning and evening and at any other time they are in pain. Using the app kids see what different types of pain they feel, what makes the pain better or worse, how it affects daily activities and what can be done to deal with the pain. The app is a helpful way for kids and their caregivers to understand their pain better. It is also a good way to share information with treating doctors so they can help manage the child’s pain better. Studies done using this app show that it is practical tool that is useful and easy to use.

For this study, we will be talking to parents or main caregivers of children with cancer and asking them how this app can be made better. We will also be talking to health care staff who provide care to children with cancer. We will then make changes to the app based on what parents and health care staff tell us, so the app is as useful as possible to parents.

**Audio recording**

The interview will audio recorded. This is being done to make sure we document your responses as accurately as possible. If you do not want to be audio recorded, you cannot be part of this study. The tape will be typed out word for word (transcribed). After the typed reports (transcripts) have been verified, the tapes will be destroyed.

**Interview preamble**

“Hello. I am part of a research team that is working on building an app to support caregivers to manage their child’s cancer pain in the home-setting. We have already developed a similar app for teens with cancer and would like to modify it so caregivers of younger children with cancer (2-11 years) can use it.

Here are some screenshots of the app for teens [show screenshots]. The app currently works by alerting teens twice daily to complete an 8-item pain report. Teens can also complete pain reports any other time during the day if they would like. Whenever a teen reports that they are having pain, the app provides instant pain self-management advice, including reminders to take pain medications and suggestions to do use physical or psychological strategies like stretching or relaxation. If a teen reports moderate to severe pain (>3/10) three times consecutively, an email is sent to a nurse at the hospital who contacts the teen’s primary oncologist and the teen to decide on a course of pain management action.

If it is ok, now I would like to ask some questions a potential new app for caregivers of younger children with pain. Please remember that you do not have to answer any of the questions are you are not comfortable with. You can also withdraw from the study at any time. If you withdraw, any data we collected from you will be safely destroyed.”

**“As I mentioned, we are interested in developing an app that would help caregivers record their child’s cancer pain and give caregivers advice on how to manage their child’s pain when it happens”**

***UTAUT2 performance expectancy*** *(“Degree to which using a technology will provide benefits to consumers in performing certain activities”) and* ***van Houtven caregiver clinical knowledge*** *(“Required skills and knowledge to carry out caregiving activities”)*

1. What are your general thoughts related to the idea of an app-based tool to support caregivers to assess and manage pain in children 2-11 years of age with cancer?
2. What do you like best about the idea of this app?
3. What do you like least about the idea of this app?
4. Do you think this app would be a useful tool for caregivers to record and manage their child’s pain?
   1. Why or why not?
5. Do you think data recorded by caregivers would be useful to you in managing the child’s cancer-related pain?
   1. Why or why not?
6. If this app could give caregivers pain management advice when they reported their child had pain, how confident are you that caregivers could successfully carry out the advice?
7. What would make you more or less confident?

***UTAUT2 effort expectancy*** *(“Degree of ease associated with consumers’ use of technology”) and* ***van Houtven quantity of caregiving*** *(“Objective burden, including the time spent caregiving”)*

1. Do you predict any difficulties with caregivers using the app?
   1. Technical problems with their phone?
   2. Not knowing how to use the app?
   3. Not understanding the text in the app?
   4. Not knowing when to use the app with their child?

***UTAUT2 social influence*** *(“Extent to which consumers perceive that important others believe they should use a particular technology”) and* ***van Houtven support seeking skills*** *(“developing organizational, tactical, and recruiting skills that help support caregiving efforts”)*

1. When should the child’s healthcare team help caregivers at home manage your child’s pain?
2. What are your thoughts about having a nurse review pain recorded by caregivers on the app from the hospital and then call the family to help with pain management?
3. How severe should a child’s pain be at home before the healthcare team should be alerted to it?

***UTAUT2 facilitating conditions*** *(“consumers’ perceptions of the resources and support available to perform a behavior”) and* ***van Houtven psychological skills and resources*** *(“coping and self-efficacy enhancing skills for caregivers”)*

1. Now that you have an idea of the app we want to build, do you have any suggestions for the app?
   1. Can you tell me more about that?
2. What questions do you think the app should ask caregivers as part of a pain assessment for their child?
   1. Pain severity?
   2. Pain interference with things like their child walking or eating?
   3. Pain interference with things like their child playing?
   4. Pain interference with things like their child sleeping?
   5. Pain location?
   6. Pain bother?
   7. Anything else?
3. What sort of pain management advice should the app provide caregivers to support them in treating their child’s pain?
4. Should the app help caregivers make choices about when to give pain medications? Can you tell me more about that?
5. Should the app help caregivers make choices about when to do physical strategies with their child like exercising or stretching? Can you tell me more about that?
6. Should the app help caregivers make choices about when to do psychological strategies

***UTAUT2 hedonistic motivation*** *(“The fun or pleasure derived from using a technology”)*

No questions.

***UTAUT2 habit*** *(“The extent to which people tend to perform behaviors automatically because of learning”)*

12. How many times a day should caregivers use the app to record their child’s pain?

1. Do you think using the app once or twice a day is a realistic thing for caregivers to do? Can you tell me more about that?

**Closing question**

Is there anything else you would like to mention about cancer pain or the app?

**Interview conclusion**

“Thank you very much for participating in this study. The information you have provided is invaluable to use as well refine and develop an app for parents providing pain management to their young children with cancer.”
